# How Gait Nonlinearities in Individuals Without Known Pathology Describe Metabolic Cost During Walking Using Artificial Neural Network and Multiple Linear Regression

**DOI:** 10.3390/app142311026

**Published:** 2024-11-27

**Authors:** Arash Mohammadzadeh Gonabadi, Farahnaz Fallahtafti, Judith M. Burnfield

**Affiliations:** 1 Institute for Rehabilitation Science and Engineering, Madonna Rehabilitation Hospitals, Lincoln, NE 68506, USA; 2 Department of Biomechanics and Center for Research in Human Movement Variability, University of Nebraska at Omaha, Omaha, NE 68182, USA

**Keywords:** nonlinearities, metabolic cost, artificial neural networks, multiple linear regression, biomechanics, gait, human movement analysis

## Abstract

This study uses Artificial Neural Networks (ANNs) and multiple linear regression (MLR) models to explore the relationship between gait dynamics and the metabolic cost. Six nonlinear metrics—Lyapunov Exponents based on Rosenstein’s algorithm (LyER), Detrended Fluctuation Analysis (DFA), the Approximate Entropy (ApEn), the correlation dimension (CD), the Sample Entropy (SpEn), and Lyapunov Exponents based on Wolf’s algorithm (LyEW)—were utilized to predict the metabolic cost during walking. Time series data from 10 subjects walking under 13 conditions, with and without hip exoskeletons, were analyzed. Six ANN models, each corresponding to a nonlinear metric, were trained using the Levenberg–Marquardt backpropagation algorithm and compared with MLR models. Performance was assessed based on the mean squared error (MSE) and correlation coefficients. ANN models outperformed MLR, with DFA and Lyapunov Exponent models showing higher R^2^ values, indicating stronger predictive accuracy. The results suggest that gait’s nonlinear characteristics significantly impact the metabolic cost, and ANNs are more effective for analyzing these dynamics than MLR models. The study emphasizes the potential of focusing on specific nonlinear gait variables to enhance assistive device optimization, particularly for hip exoskeletons. These findings support the development of personalized interventions that improve walking efficiency and reduce metabolic demands, offering insights into the design of advanced assistive technologies.

## 1. Introduction

### 1.1. Introduction to Artificial Neural Networks (ANNs) in Biomechanics

Artificial Neural Networks (ANNs) are computational models inspired by the human brain’s structure and function [1,2]. Our previous research has explored a wide range of Artificial Intelligence (AI) applications, showing how optimization methods and ANNs can be effective in tackling challenges across different fields [3–8]. These studies highlight the adaptability of AI techniques and provide new visions for biomechanical analysis. In biomechanics, ANNs have been applied to analyze and interpret complex biological rhythms [9,10], model human locomotion [11], and predict injury risks and athletic performance [12]. Recent studies have used ANNs to understand movement mechanics better and predict metabolic costs [13,14], showcasing substantial advancements in personalized healthcare and athletic performance optimization. These applications highlight the adaptability of AI techniques in biomechanical analysis and their potential for facilitating targeted interventions and assistive technologies (e.g., lower extremity orthoses) to improve movement efficiency and lower the risk of injury.

### 1.2. ANN Applications and Nonlinear Measures in Gait Analysis

Our study outlines an innovative approach to understanding the intricacies of gait dynamics and their metabolic implications through the lens of ANNs and nonlinear analysis. Nonlinear measures quantify human locomotion’s complex and dynamic nature and have the potential to offer insights that would be invaluable for customizing assistive device settings to improve walking. Six nonlinear metrics relevant to the examination of gait variability and complexity are studied: the Lyapunov Exponent using the Rosenstein algorithm (LyE_R_), Detrended Fluctuation Analysis (DFA), the Approximate Entropy (ApEn), the correlation dimension (CD), the Sample Entropy (SpEn), and the Lyapunov Exponent using the Wolf algorithm (LyE_W_).

These measures provide a nuanced view of human locomotion, highlighting the unpredictable and complex nature of gait patterns that traditional linear analyses might overlook. For example, the Lyapunov Exponent (LyE) is critical in analyzing dynamic systems, including the stability and variability of walking [15]. The LyE measures the local divergence from an attractor, such as a steady state condition, thereby quantifying the motor system’s capacity to dampen minor disturbances [16]. The algorithms of Wolf et al. (LyE_W_) [16–19] and Rosenstein et al. (LyE_R_) [15,20] each apply unique normalization processes to the time series data [15]. The DFA is used to analyze time series data to identify long-range correlations [21–26]. It employs the fractal scaling index, alpha. In gait analysis, an alpha of 0.5 to 1.0 indicates statistical persistence in the intervals between steps, meaning these intervals are consistent over a long distance, with minor fluctuations corrected in subsequent strides. Conversely, an alpha value below 0.5 denotes anti-persistence, where intervals vary and appear randomly over a long range, with immediate adjustments for deviations.

The ApEn [27–30] quantifies regularity and unpredictability in time series data. The ApEn is particularly useful in analyzing complex systems where data patterns are not immediately apparent, such as subtle changes in the inherent repeatability of cyclic gait patterns, which may reflect underlying pathology. High ApEn values suggest greater system complexity and less predictability, whereas lower values indicate more regular and predictable patterns. The CD, which has been used to study the complexity of gait patterns between different populations [31], elucidates how data points from a dynamical system, such as a kinematic time series from walking, are arranged within a state space. Small correlation dimension values suggest a limited number of available degrees of freedom, while values in the range of 1.5 to 2.5 are typically associated with deterministic data. In contrast, large values (e.g., 4.0 to 6.0) indicate entirely random data. The SpEn, the final nonlinear measure studied, is less dependent on the data series length and displays a greater capacity to distinguish between similar and dissimilar patterns in a dataset than the ApEn (e.g., single- and dual-task conditions) [32,33].

### 1.3. Study Focus on Exoskeleton-Assisted Walking

The current research leverages ANNs to delve deeper into the biomechanics of movement, particularly in exoskeleton-assisted walking. By analyzing kinematic, kinetic, and metabolic data from subjects walking under various conditions with assistance, the study evaluates the effectiveness of ANNs in capturing and predicting the metabolic cost associated with different gait patterns. This is compared against traditional multiple linear regression (MLR) models to assess their relative predictive capabilities.

### 1.4. Hypotheses

Our first hypothesis indicates that the metabolic cost of gait is intricately linked to its nonlinearity, suggesting that the complex dynamics of walking influence energy expenditure. Our second hypothesis contends that due to their proficiency in capturing complex, nonlinear patterns, ANNs will offer a more precise and reliable method for analyzing the metabolic implications of gait dynamics compared to MLR models.

The implications of this study represent an initial step toward enhancing assistive device optimization, particularly in applications such as exoskeleton design. The study provides valuable insights into improving assistive devices and tailoring interventions to meet individual needs by exploring more detailed gait analysis and rehabilitation techniques. Enhancing our comprehension of gait’s nonlinear characteristics and their metabolic effects could improve the quality of life for those with walking limitations while also making substantial contributions to the expansive domains of biomechanical analysis and the development of assistive technologies.

### 1.5. Study Objectives

The primary objectives of this study are to (1) develop and evaluate MLR and ANN models for predicting the metabolic cost during gait based on nonlinear gait measures, (2) identify key nonlinear metrics, such as the LyE_R_, DFA, ApEn, CD, SpEn, and LyE_W_, that are most effective in capturing gait dynamics related to the metabolic cost, and (3) compare the performance of MLR and ANN models to determine their strengths and limitations in handling nonlinear gait data. This study aims to contribute to designing adaptive, real-time gait analysis systems and potential exoskeleton technology applications.

## 2. Materials and Methods

### 2.1. Biomechanical and Metabolic Data

This study involved a secondary analysis of a previously reported dataset focused on the impact of a bilateral semi-rigid hip exoskeleton on gait characteristics [34]. The hip exoskeleton was a semi-rigid model designed to assist hip extension with a temporal force-tracking controller that applied sinusoidal torque profiles at specified times during the stride cycle [34]. This controller independently adjusted torque profiles for each leg. The torque assistance ranged in magnitude, with peak torques of 0.06 to 0.12 N·m/kg. The desired timing for assistance termination varied from 21% to 49% of the gait cycle. This variable end timing and magnitude allowed us to explore the impact of assistance timing on reducing the metabolic cost [34]. The exoskeleton interfaced with participants via a waist belt and thigh cuffs positioned above the knees, allowing for targeted torque application across the hip joint. Adjustable straps ensured a secure, customized fit, and the device’s lightweight, flexible design allowed for natural hip flexion and extension with minimal gait restriction. In brief, ten adults without a known disability (four males, six females; age: 27.6 ± 5.9 years; body mass: 65.3 ± 13.1 kg; height: 1.66 ± 0.08 m; BMI: 23.39 ± 4.10) enrolled in an Institutional Review Board-approved study. Participants were selected based on inclusion criteria that ensured they had no known musculoskeletal or neurological conditions that might influence gait patterns. Each performed 12 walking trials on a force plate-instrumented treadmill (Bertec, Columbus, OH, USA) at 1.25 m/s. During one trial, participants did not wear the exoskeleton. During 11 trials, participants walked while wearing a hip exoskeleton designed to assist hip motion. At the same time, the magnitude and timing of the extensor torque assistance were manipulated from no assistance to a peak torque of 0.12 (N·m/kg). Oxygen consumption and carbon dioxide production were measured using indirect calorimetry (K5, Cosmed, Rome, Italy), and breath-by-breath data in the final two minutes were used to estimate the steady-state metabolic rate under each walking condition in watts per kilogram (W/kg). Three-dimensional lower extremity kinematics were recorded (VICON Vero, Oxford Metrics, Yarnton, UK; 120 Hz) from 23 reflective markers placed on the skin, a tight-fitting suit, and the exterior surface of the shoes over anatomical landmarks. Inverse kinematics were used to estimate joint kinematics from the marker data, limiting ankle and knee motion to a single degree of freedom (flexion and extension) while allowing three degrees of freedom for the hip. Ground reaction forces (GRFs), recorded (2000 Hz) using force plates embedded in the treadmill, were normalized to each participant’s body mass (N/kg). Kinematic and kinetic data were used within OpenSim (version 4.0, SimTK, San Jose, CA, USA; [35,36]) to calculate joint moments, normalized to each participant’s body mass (N·m/kg). We used the same software for gait validation tests, a widely recognized tool for simulating and analyzing musculoskeletal dynamics.

In brief, the calibration for motion capture involved placing reflective markers on anatomical landmarks to establish a local coordinate system [20]. Before data collection, a static calibration trial was performed with the participant upright to align marker positions accurately with joint centers. For force measurements, the embedded force plates underwent zeroing and calibration with known weights to ensure precise ground reaction force readings. Calibration was repeated regularly throughout data collection to maintain accuracy and consistency in motion capture and force measurement systems. A laboratory-based global coordinate system was used, with the *x*-axis in the anterior–posterior, the *y*-axis in the medial–lateral, and the *z*-axis in the vertical directions. Transformations were applied to convert marker data from the global to each joint’s local coordinate system, ensuring accurate joint angle calculations.

Each trial captured up to 60 consecutive strides to minimize kinematic and kinetic time series interruptions. However, in cases where data gaps were present due to marker occlusion or sensor signal loss, we used interpolation techniques to fill these gaps. Specifically, the cubic spline method was employed, as it has been shown to effectively reduce errors in linear and nonlinear kinematic calculations when managing missing data points during cyclic locomotor activities [18,19]. Given the focus on nonlinear measures, no filtering was applied to the marker trajectories to preserve the original data characteristics. This approach ensured the calculated variables’ integrity and maintained the stride-averaged time series consistency across trials.

After the pre-processing of the kinematic and kinetic data, six nonlinear measures (LyE_R_, DFA, ApEn, CD, SpEn, LyE_W_) were applied to 13 gait variables to quantify the complexity, regularity, and dynamic stability of human gait patterns. Nonlinear measures were calculated using established algorithms with specific parameters. The LyE_R_ and LyE_W_ [15,20] used Rosenstein’s and Wolf’s [16–19] algorithms (embedding dimension *m* = 5, time delay *τ* = 1, [16–19]); DFA calculated a scaling exponent α over windows of 4 to 60 strides [21–26]; the ApEn [27–30] and SpEn [32] used *m* = 2 and the tolerance *r* = 0.2 × the data’s standard deviation [33]; and the CD estimated the degrees of freedom with embedding dimensions *m* = 2 to *m* = 10 [31]. Analyses were performed using MATLAB R2024b custom codes. These measures were used to understand the relationship between gait dynamics and the metabolic cost during walking, helping to identify how these nonlinear characteristics influence energy expenditure. In particular, the nonlinear value for each measure was calculated for the sagittal plane joint angles (hip, knee, and ankle), velocities (hip, knee, and ankle) and moments (hip, knee, and ankle), the position and velocity of the center of mass in the sagittal plane, and the magnitude of the GRF in the vertical and anterior–posterior directions. We focused on the sagittal plane, as it captured key joint angles, moments, and velocities most relevant to forward progression and the metabolic cost while maintaining computational efficiency, given our sample size.

### 2.2. Neural Network Design and Implementation

Our study designed and implemented ANNs to investigate the predictive power of nonlinear gait measures (LyE_R_, DFA, ApEn, CD, SpEn, LyE_W_) for metabolic cost. The process commenced by loading the nonlinear gait data and setting up the neural network parameters and architecture. The networks were trained using the Levenberg–Marquardt backpropagation algorithm [1], renowned for its efficiency in solving nonlinear problems [2]. We chose ANNs over other machine learning models due to their effectiveness in capturing the complex, nonlinear relationships inherent in gait dynamics, which are key to our analysis [14,15,37]. Moreover, studies in biomechanics demonstrate their utility with small datasets [10,14,15]. Additionally, ANNs are well established in gait and exoskeleton research, offering reliable performance in predicting movement-related outcomes, further supporting our choice for this specific application [10,14,15]. The data division for training, validation, and testing was set at 70%, 15%, and 15% of the dataset to ensure a balanced approach to learning and generalization. A subject-wise splitting strategy was applied, ensuring all trials from a single subject were included exclusively in the training, validation, or testing set. This prevented data leakage and ensured the model was tested on entirely new subjects, enhancing generalizability. The network’s performance was closely monitored throughout the training process, focusing on the error distribution, response correlation, and predictive accuracy across the training, validation, and testing phases. This involved analyzing prediction errors and their correlation with input variables, offering insights into the network’s behavior and areas for improvement. Regression analysis was employed to quantify the alignment between the network’s outputs and the actual metabolic rates, offering a comprehensive evaluation of the model’s effectiveness. The networks were trained, validated, and tested using a subset of the dataset, with performance metrics such as the mean squared error (MSE) and correlation coefficients (R-values) as key evaluation criteria [37]. This methodical approach to ANN design and implementation enabled us to harness the power of neural networks by analyzing complex biomechanical data to enhance our understanding of the relationship between gait nonlinearities and metabolic cost.

### 2.3. Neural Network Optimization Process

Our model included 13 input features derived from nonlinear gait metrics and a single output node representing the predicted metabolic cost. Given the small sample size, we used an ANN instead of deep learning [10,14]. To improve the reliability of our neural network models and balance the convergence speed and stability within our smaller dataset [14,38], we implemented a comprehensive optimization approach inspired by Nogueira et al. [38] and Gonabadi et al. [14]. We applied both grid search and random search techniques to systematically identify the optimal hidden layer sizes for each ANN model [14,38]. The final selection was based on performance metrics, specifically the mean squared error (MSE) and R-values, evaluated across cross-validation folds, ensuring that the hidden layer sizes achieved a balance between the model complexity and predictive accuracy [14,38]. This approach utilized nested for loops to systematically examine different network configurations, varying the hidden layer size (from 1 to 50) to assess the impact of the network complexity on accuracy and performing 1000 iterations for each parameter combination to ensure stability and reduce stochastic variations. Each configuration was evaluated using predefined metrics, including the MSE for training, validation, and test sets, along with correlation coefficients (R-values) to assess predictive accuracy. This systematic approach ensured that the final models were optimized for the network architecture and consistently delivered reliable performance across iterations. We observed that as the number of individual neurons within a single hidden layer increased, the model performance initially improved, reflected by decreases in the MSE and increases in R-values, indicating better predictive accuracy. However, beyond a certain complexity threshold, additional neurons led to diminishing returns and, in some cases, slight overfitting, as indicated by the increased MSE in the validation set. This analysis helped identify an optimal architecture that balanced complexity with predictive reliability. The entire optimization procedure was conducted using MATLAB (MathWorks, Natick, MA, USA), leveraging its Neural Network Toolbox for building and training the models. Additionally, we crafted customized MATLAB codes specifically for this research.

### 2.4. Data Integration and Analysis

Considering the diverse origins of the data, a significant effort was dedicated to merging the varied time series data into a unified dataset suitable for analysis by the ANN models. This unification process included standardizing data formats, synchronizing time series from various trials according to phases of the gait cycle, and normalizing the data to minimize differences between subjects. When synchronizing the time series from various trials, we aligned the initial contact of the dominant limb across trials rather than adjusting the temporal length of individual gait cycles. This approach ensured that all gait cycles began consistently without causing the elongation or shortening of data segments [14]. Normalization was performed to minimize differences between subjects and ensure the comparability of input variables for the ANN model. Table 1 shows all measured signals and their corresponding normalization processes. To account for differences in body anthropometrics across participants, the joint moments and GRF were normalized to each participant’s body mass (N·m/kg for moments and N/kg for the GRF), while the metabolic cost was expressed in watts per kilogram (W/kg) [14]. The resulting dataset, encompassing 120 trials, formed an extensive collection of biomechanical variables crucial to the aims of our research.

### 2.5. Partial Dependence Plots (PDPs)

Partial Dependence Plots (PDPs) are a crucial instrument for elucidating the dynamics within machine learning models, particularly in understanding how different predictors influence the predicted outcome [39–41]. These plots offer a visual interpretation of the effect each predictor variable—such as joint angles, velocities, moments, the center of mass’s position and velocity in the sagittal plane, and the GRF in both vertical and anterior–posterior directions—has on the response variable, which in this context was the metabolic cost associated with gait. By systematically varying the value of one predictor while holding others constant, PDPs enable us to observe the impact of that predictor on the Root Mean Square Error (RMSE) of the model’s predictions. This approach helped pinpoint the variables that significantly influence the model’s accuracy and, by extension, the metabolic cost predictions. The resulting plots provided a graphical representation of the relationship between each variable and the predicted metabolic cost. They illustrated how changes in a specific variable are associated with variations in prediction accuracy, illuminating the crucial variables essential for the accurate estimation of the metabolic cost. Employing PDPs across different models that leverage gait nonlinearities allowed for a deeper understanding of which gait parameters are crucial for accurately predicting metabolic expenditure during walking.

### 2.6. Statistical Analyses

In our investigation of the effectiveness of nonlinear measures (LyE_R_, DFA, ApEn, CD, SpEn, LyE_W_) for predicting the metabolic cost, we constructed six unique ANNs. These ANNs underwent a thorough evaluation to determine their capacity as predictors of metabolic expenditure, comparing their performance against traditional multiple linear regression (MLR) models for each nonlinear metric. The assessment of the ANNs encompassed an analysis of the error distribution, response correlation, and predictive accuracy during various data phases, including training, validation, and testing. Additionally, we scrutinized the patterns in prediction errors and their association with input variables to identify the models’ strengths and areas requiring enhancement. We expanded our assessment metrics to include 5-fold cross-validation (k = 5) for a comprehensive evaluation of the ANN models, which provided more reliable metrics and minimized overfitting, particularly given the small dataset [14,42]. Regression analysis was employed to measure the alignment of the model outputs with the actual target values, providing a detailed understanding of the model’s predictive capabilities. In addition, the regression analyses underscore the complexity of predicting the metabolic cost from gait variables. The differences in the R^2^_ANN_ and R^2^_MLR_ values and the significance of various predictors across the nonlinear measures highlight the nuanced relationship between gait dynamics and the metabolic cost. These insights are crucial for developing more accurate models for metabolic cost prediction. To provide a more detailed evaluation of model performance, we conducted supplementary analyses, including error histograms and scatter plots comparing predicted and actual values and confidence intervals across nonlinearity measures. These Supplementary Figures and Table are included in the Supplementary Materials and offer additional insights into the distribution of errors and the prediction accuracy. We encourage readers to refer to these figures to understand the model’s performance across different gait conditions comprehensively. This comprehensive approach enabled us to leverage sophisticated nonlinear analyses to dissect the complex biomechanical data from hip exoskeleton-assisted walking. We aimed to derive profound insights into assisted gait dynamics and formulate an improved and precise predictive framework for assessing the metabolic implications of hip exoskeleton use by contrasting the predictive abilities of ANNs with MLR models. All statistical procedures were conducted using MATLAB, with a set significance threshold of 0.05 for all tests. Model performances were compared using the MSE and R-squared values. The *p*-values reported in Table 2 were derived from the MATLAB “*corrcoef*” function, which calculates the R^2^ and a two-tailed *p*-value for each dataset (training, validation, and testing) [10,14,43]. These *p*-values test the null hypothesis, in particular, that there is no linear relationship between the predicted and actual values [10,14,43]. The extremely low *p*-values observed reflect a statistically significant correlation, validating the model’s predictive accuracy [10,14,43].

Based on the literature and similar studies [1,4,10,14,37,38,43], we ensured that the statistical methods applied in this study were appropriate for the structure of our dataset. These adjustments align with the recommendations provided in prior studies that analyzed similar datasets.

**Figure 1.**
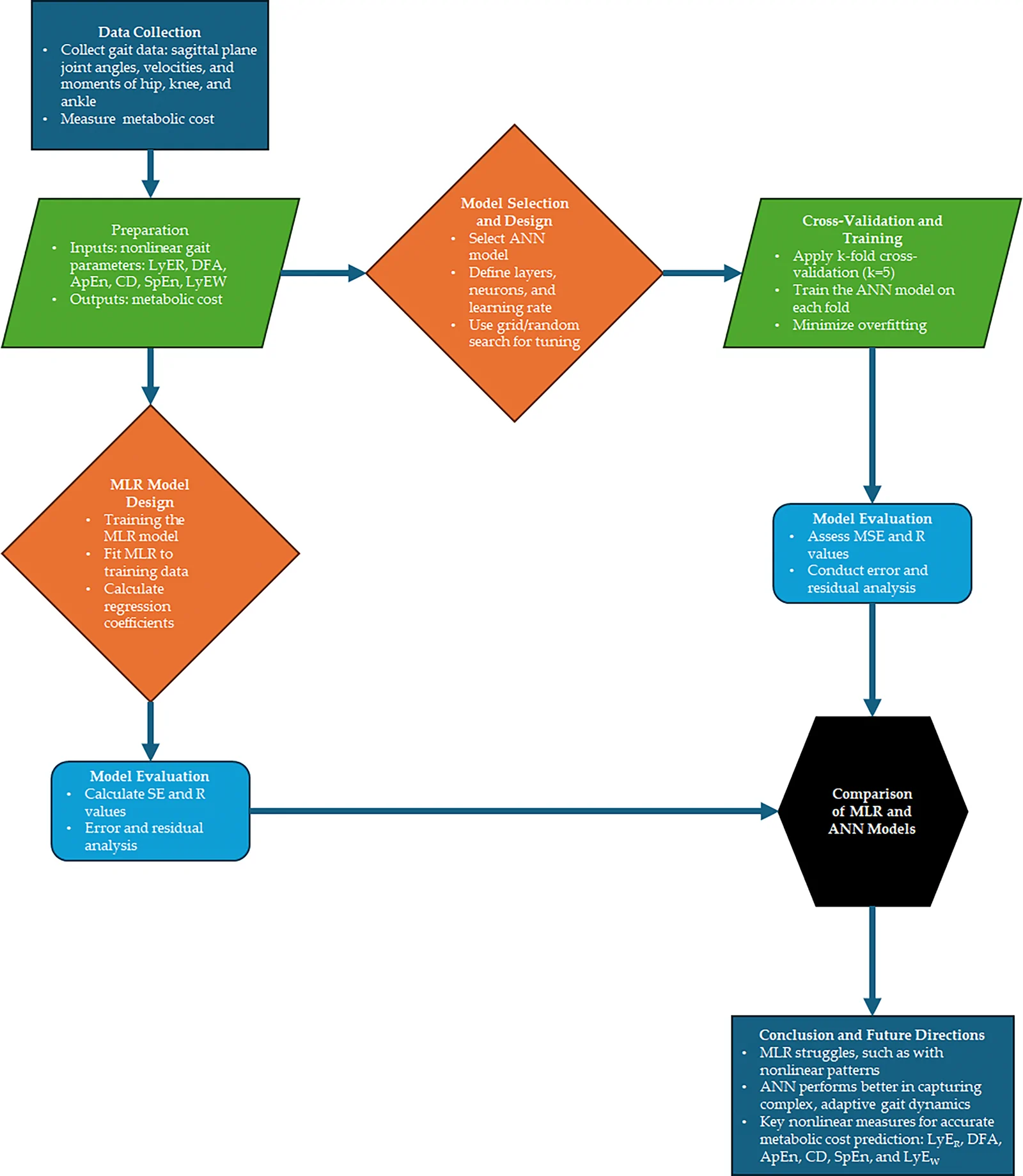
Flow diagram of the research development process for predicting the metabolic cost using multiple linear regression (MLR) and Artificial Neural Network (ANN) models, based on six gait nonlinearity measures: the Lyapunov Exponent based on Rosenstein’s algorithm (LyER), Detrended Fluctuation Analysis (DFA), the Approximate Entropy (ApEn), the correlation dimension (CD), the Sample Entropy (SpEn), and the Lyapunov Exponent based on Wolf’s algorithm (LyEW). The diagram outlines the sequential steps, from data collection and preparation through model design, cross-validation, and evaluation, and a comparative analysis of ANN and MLR models. Each variable represents specific gait parameters, including joint angles, velocities, moments, ground reaction forces (GRFs), and center of mass (COM) metrics. Key nonlinear measures for accurate metabolic cost prediction are emphasized, along with conclusions on the strengths and limitations of each model.

Figure 1 illustrates the research process, including each step involved in developing and evaluating the MLR and ANN models for predicting the metabolic cost. This visual highlights key nonlinear measures identified for accurate metabolic cost prediction, as well as insights into the strengths and limitations of each model.

## 3. Results

Six unique ANN models were constructed, each focusing on a different gait nonlinearity measure (LyE_R_, DFA, ApEn, CD, SpEn, LyE_W_) derived from the kinematic and kinetic features calculated across the 120-trial dataset to estimate metabolic costs.

Table 2 and Supplementary Figures show the performance of six different ANN models, each tailored to a specific gait nonlinearity measure (LyE_R_, DFA, ApEn, CD, SpEn, LyE_W_). The varied number of neurons in the hidden layer across models suggests that differing levels of complexity are required to capture best the relationship between each nonlinearity measure and the metabolic cost. Models with the highest number of neurons reflected the greatest complexity, while those with the fewest neurons reflected the least complexity. Performance was evaluated using the mean squared error (MSE) and R^2^_ANN_, with lower MSE and higher R^2^_ANN_ values indicating better model performance. The *p*-values assessed the statistical significance of the model’s predictions. Supplementary Table represents the confidence intervals for the MLR and ANN models to provide a more robust assessment of the model reliability.

Tables 3 and 4 present the results of multiple linear regression analyses for various nonlinear gait measures: the LyE_R_, DFA, ApEn, CD, SpEn, and LyE_W_. The R^2^_MLR_ values indicate the proportion of variance in the metabolic cost explained by each model, with the LyE_R_ and DFA showing the highest explanatory power at 0.72 and 0.63, respectively. Every row in the tables corresponds to a distinct gait parameter, including the angles, velocities, and moments of the hip, knee, and ankle joints, along with the velocity and position of the COM and the anterior–posterior and vertical components of the GRF. The coefficients (estimates) reflect the change in the metabolic cost associated with a one-unit change in the variable, holding other variables constant. The Standard Error (SE) measures the variability in the estimate, and the *p*-value indicates the statistical significance of the coefficients.

**Figure 2.**
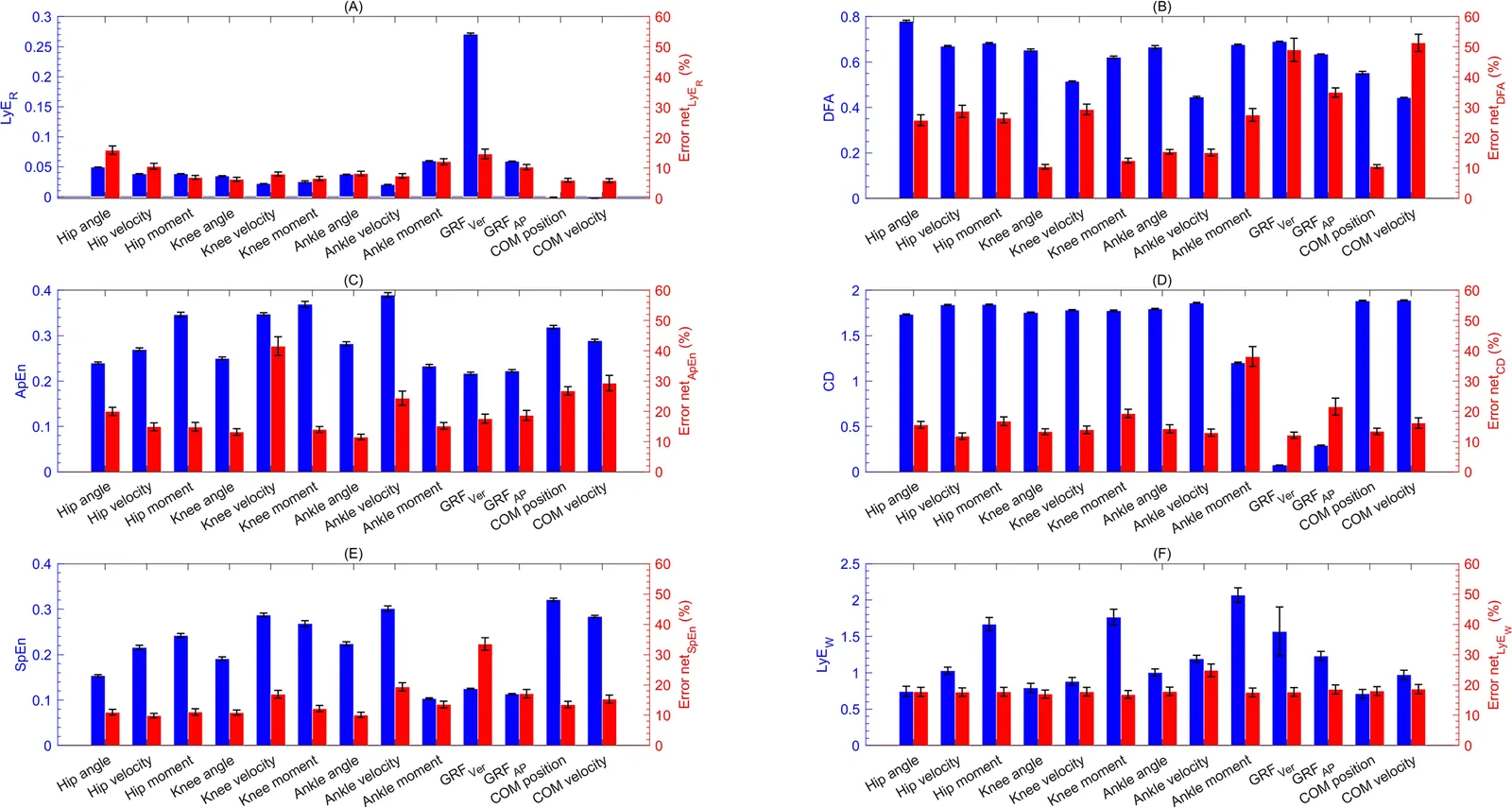
Partial Dependence Plots (PDPs), the graphical analysis of gait nonlinearity measures, and their prediction errors. This figure illustrates the relationship between various gait parameters—such as joint angles, velocities, moments, center of mass (COM) displacement in the sagittal plane, and ground reaction force (GRF) magnitudes in vertical and anterior–posterior directions—and their influence on the prediction of the metabolic cost. Subfigures represent the mean of nonlinearity measures, (**A**) the Lyapunov Exponent based on Rosenstein’s algorithm (LyE_R_), (**B**) Detrended Fluctuation Analysis (DFA), (**C**) the Approximate Entropy (ApEn), (**D**) the correlation dimension (CD), (**E**) the Sample Entropy (SpEn), and (**F**) the Lyapunov Exponent based on Wolf’s algorithm (LyE_w_), respectively. Blue bars (left vertical axis) indicate the measure values, while red bars (right vertical axis) show the corresponding prediction error of energy expenditure percentages, highlighting the impact of each gait parameter on the precision of metabolic cost estimation.

This study employed Partial Dependence Plots (PDPs) to delve into the influence of specific gait variables—such as joint angles, velocities, moments, the center of mass position and velocity in the sagittal plane, and the GRF in vertical and anterior–posterior directions—across different nonlinear analyses on metabolic cost estimation [39–41]. PDPs were utilized for each nonlinear measure to identify variables that significantly impact the model’s predictive accuracy, as reflected by the RMSE. For each model, individual variable signals were adjusted through the entire range of their observed values during the gait cycle while keeping everything else constant. Changes in the RMSE arising from the manipulation of each variable enabled the determination of the gait parameters that are most crucial for precise metabolic cost predictions. Figure 2 represents the graphical analysis of gait nonlinearity measures and their prediction errors. Table 5 represents the summary of nonlinear gait parameters and ANN prediction errors.

## 4. Discussion

This study explored the nonlinear dynamics of human gait and their correlation with the metabolic cost using ANNs. It assessed the predictive capabilities of ANNs compared to traditional MLR models across a spectrum of nonlinear gait measures.

In the discussion of the comparative analysis of the ANN models, each tailored to a specific gait nonlinearity measure (LyE_R_, DFA, ApEn, CD, SpEn, LyE_W_), we observe a spectrum of performance metrics that highlight the complexity of estimating the metabolic cost using models based on the nonlinearity of gait measures. The performance metrics in Table 2 and Supplementary Figures demonstrate the training, validation, and test splits and the hidden layer sizes for each ANN model. These models were meticulously developed to capture the intricate relationships between the nonlinear gait measures and metabolic cost effectively. The training performance across all models was robust, with high R^2^_ANN_ values close to one, indicating an excellent fit [44]. Specifically, the LyE_R_, DFA, and ApEn models showed superior performance, with low MSE and high R^2^_ANN_ values. These measures could capture the inherent dynamics in the gait pattern, which correlate with the metabolic cost more effectively than other measures. The results in Table 1 of the k-fold cross-validation show consistent trends in the ANN R_ANN_ and *p*-values, similarly to the ‘Total’ values calculated using combined data from the training, validation, and test phases. This approach offers a more robust model evaluation, particularly with a small sample size, as it provides more reliable metrics and reduces the risk of overfitting. Significant *p*-values were observed across all model assessment phases (validation, test, and training), confirming the robust predictive capabilities of the models. For example, the LyE_R_ reflects the system’s sensitivity to small perturbations, potentially indicating stability and control adjustments in gait that directly impact energy expenditure. DFA captures long-range correlations in gait patterns, representing sustained coordination efforts that may increase metabolic demands. The ApEn quantifies the complexity of gait variability, which could relate to the adaptability of gait mechanics in response to changing conditions. In contrast, while still yielding statistically significant results, models such as LyE_w_ presented lower R^2^_ANN_ values, especially in the validation phase, which could indicate a less generalizable model.

To provide additional insights into the practical significance of the results, we have classified the strength of the correlations observed in this study using Cohen’s guidelines [45–49] for effect size magnitudes: trivial (<0.10), small to medium (0.10–0.30), medium to large (0.30–0.50), and large to very large (>0.50) [45–49]. These classifications, combined with Pearson’s R-values and R^2^ values reported in Tables 2–4, offer a comprehensive understanding of the strength and relevance of the relationships modeled. This approach highlights both statistical significance and practical importance within the context of our study.

The varied hidden layer sizes (individual neurons within a single hidden layer) across models reflect the individual complexities of each nonlinear measure; the selection of specific neuron counts was based on iterative testing during the model optimization process. For instance, the LyE_w_ and CD with minimal hidden layer sizes of two and three neurons indicate a more straightforward model structure. The CD and LyE_w_ capture key aspects of system dynamics. The CD reflects dimensionality or complexity (degrees of freedom) [31], while the LyE_w_ measures sensitivity to initial conditions, indicating stability [16–19]. These metrics provide relatively direct information on complexity and stability, likely requiring fewer individual neurons within a single hidden layer in the neural network to model accurately. Their consistency across variables shown in Figure 2 also introduces less noise, allowing for reliable predictions with simpler architectures. In contrast, DFA, with 19 neurons, indicates a need for a more complex model, as it captures long-range correlations and intricate, nonlinear relationships within the data [21–26]. This added complexity reflects the raw variability in values shown in Figure 2, requiring more neurons to capture subtle patterns in gait variability effectively. DFA with 19 neurons suggests a more complex model is needed to map the measure to the metabolic cost accurately. Overall, the ANN models demonstrated a substantial capacity for predicting the metabolic cost from nonlinear gait measures. However, differences in performance metrics such as MSE and R^2^_ANN_ values underscore the importance of model selection based on the specific nonlinear feature being analyzed. These insights are crucial for further refining predictive models and potentially translating these findings into developing advanced assistive technologies.

Supplementary Figures display the error histograms with 20 bins, which graphically represent the distribution of errors in the set of all six nonlinear ANN and MLR model predictions. The *x*-axis shows the error magnitude, with negative values representing underpredictions and positive values representing overpredictions relative to the actual nonlinear ANN model values. The *y*-axis shows the number of instances (frequency) of each error magnitude in the dataset. The error histograms in Supplementary Figures provide a comprehensive overview of the performance of six different nonlinear ANN and MLR models. These models, namely net_LyER_, net_DFA_, net_ApEn_, net_CD_, net_SpEn_, and net_LyEw_ for the ANN models and MLR_LyER_, MLR_DFA_, MLR_ApEn_, MLR_CD_, MLR_SpEn_, and MLR_LyEw_ for the MLR models, are evaluated based on their prediction errors relative to actual metabolic costs. The histograms collectively demonstrate a spectrum of accuracy and bias across models. The net_SpEn_ model displays a bell-shaped distribution peaking near zero error, reinforcing its predictive precision.

On the contrary, net_CD_ and net_DFA_ both exhibit slight underprediction biases, although their errors are primarily close to zero, suggesting overall accurate models. Net_LyEw_ is notable for its errors symmetrically distributed about the zero-error line, highlighting an unbiased model. Lastly, net_LyER_’s histogram, while still reflecting balanced predictive capabilities, slightly leans towards overpredictions. In the error histograms for the MLR models (Supplementary Figures), none of the models display a symmetrical error distribution around the zero line. Although the LyE_R_ model shows a slight trend toward symmetry near zero, it does not exhibit a distinct, bell-shaped distribution. This lack of symmetry across the error distributions suggests that the MLR models struggle to capture the underlying dynamics fully, particularly in cases with higher nonlinearity. These visual error distributions are critical in assessing and refining each model’s ability to accurately predict metabolic costs, with a tighter clustering of errors around zero indicating a higher model accuracy.

Tables 3 and 4 comprehensively overview the multiple linear regression analyses performed on various nonlinear gait measures to predict the metabolic cost. The tables summarize the regression analysis results for the six different nonlinear measures. The R^2^_MLR_ values in these tables represent how much variance in the metabolic cost can be explained by the models created using these nonlinear measures. Specifically, the LyE_R_ and DFA demonstrate the highest explanatory power with R^2^_MLR_ values of 0.72 and 0.63, respectively. This implies that these models can explain 72% and 63% of the variance in the metabolic cost, indicating a substantial connection between the predicted metabolic cost and the variables included in these models.

Each row in the tables corresponds to a different gait parameter. The coefficients, denoted as estimates, indicate how a one-unit change in each variable would impact the metabolic cost, assuming other variables remain constant. The Standard Error (SE) gives a sense of the estimate’s precision, and the *p*-values test the statistical significance of each coefficient. Regarding statistical significance, values with significant *p*-values (less than 0.05) are highlighted in bold, underscoring their strong influence on the predicted metabolic cost. Tables 3 and 4 show that the hip moment and both components of the GRF have significant coefficients, indicating that they are important predictors of the metabolic cost in the ApEn and SpEn models. In Tables 3 and 4, the significant negative coefficients for the hip moment and positive coefficients for the ankle velocity and GRF vertical component in the DFA model suggest that these factors are particularly influential in predicting the metabolic cost using this measure. Key predictors for the LyEw and LyER models were the hip and ankle velocity.

Based on Tables 3 and 4, we can identify the most influential variables in the regression analyses for predicting the metabolic cost from various nonlinear gait measures, mainly focusing on the LyE_R_ and DFA due to their higher explanatory power (R^2^_MLR_ values of 0.72 and 0.63, respectively). In Tables 3 and 4, GRF_Ver_ and GRF_AP_ have significant *p*-values across the ApEn, CD, and SpEn models, indicating their strong influence on the metabolic cost. For instance, GRF_Ver_ has a coefficient of 0.3785 with a *p*-value of 0.0004 in the ApEn model, and GRF_AP_ has a coefficient of −0.4155 with a *p*-value of 0.0003, suggesting that these GRF components are crucial predictors within these models. In the DFA model, variables like the ankle velocity and ankle moment have significant *p*-values and substantial coefficients, indicating their impactful role. Specifically, the ankle velocity has a coefficient of 0.3245 with a *p*-value of <0.0001, marking it as a key variable in the DFA model for predicting the metabolic cost. For the LyE_R_ model, the hip velocity and hip moment stand out with significant *p*-values and large coefficients, suggesting their critical influence on metabolic cost predictions. The hip velocity has a coefficient of 1.2900 with a *p*-value of 0.0002, and the hip moment has a coefficient of −1.7930 with a *p*-value of 0.0003, highlighting their importance in the LyE_R_ model.

Nonlinear analysis tools serve distinct purposes for different gait metrics. For instance, using the DFA on the ankle moment time series uncovers the neuromuscular system’s complexity and strategies for joint stability, adaptation to varied walking or running conditions, and injury risk through abnormal conditions. On the other hand, the DFA on GRF data sheds light on the fluctuation in external forces crucial for movement. While ankle moment analysis delves into joint mechanics and control, GRF analysis provides a broader perspective on the body’s interaction with its environment, which is crucial for evaluating gait stability and efficiency.

To deepen the biomechanical understanding of our ANN results, we interpret the role of specific nonlinear measures, such as DFA and the LyE_w_, in capturing the fundamental dynamics of exoskeleton-assisted gait. DFA applied to the ankle moment provides insights into the neuromuscular complexity and joint stability strategies required to adapt to external support. This adaptability is essential for preventing joint strain and ensuring stability during varied gait conditions, where subtle, complex control adjustments are needed. Similarly, the DFA on GRF data highlights the fluctuation in external forces, emphasizing the body’s interaction with its environment, which is central to maintaining movement stability and efficiency. In overall observations, GRF components seem consistently significant across models, especially in Tables 3 and 4, indicating their general importance in predicting the metabolic cost. In the LyE_R_ and DFA models, variables related to hip and ankle movements (velocity, moment) are particularly influential, suggesting that specific lower limb dynamics aspects are crucial for metabolic cost estimation. These findings highlight the complexity of gait dynamics and their relationship with the metabolic cost. Variables related to ground reaction forces and specific joint dynamics (especially at the hip and ankle) are significant predictors, underscoring the importance of these factors in the context of gait analysis and metabolic cost estimation.

Figure 2 and Table 5 offer a detailed summary of various nonlinear gait measures and their corresponding prediction errors from ANNs for a range of gait parameters. Regarding nonlinearity, the greatest value for the ApEn was observed for the ankle velocity (0.390), indicating the highest complexity, and the lowest ApEn was related to the hip angle (0.240), suggesting a more predictable pattern. The SpEn follows a similar trend, with the ankle velocity again showing the greatest (0.301) and the ankle moment the lowest unpredictability (0.103). The COM velocity presented the highest value for the CD (1.888), which signifies a complex dynamic, while the GRF_Ver_ has the lowest (0.075), implying a more straightforward structure. The highest DFA values were observed for the vertical GRF_Ver_ at 0.691 and the lowest for the COM velocity at 0.444. This indicates that the dynamics of vertical GRFs during gait may be more persistent than those of COM displacement. The LyE_w_ of the ankle moment was highest among other gait measures (2.068), reflecting significant sensitivity to initial conditions, while the hip angle exhibited the least sensitivity (0.745). High LyE_w_ values for the ankle moment indicate its sensitivity to initial conditions, reflecting the ankle’s responsiveness in maintaining propulsion and balance, especially in exoskeleton-assisted gait. The lower LyE_w_ values for the hip angle highlight the unique control demands placed on the ankle compared to other joints during assisted walking. This sensitivity to initial conditions indicates that small variations at the start of the stride cycle can lead to significant differences in gait patterns, particularly for the ankle moment. In contrast, the hip angle’s lower sensitivity suggests more consistent and stable behavior throughout the gait cycle, making it less influenced by small changes at the beginning.

In contrast, the LyE_R_ of the GRF_Ver_ (0.271) was the highest. The LyE_R_ of the COM displacement and COM velocity showed negative values. A negative value of a Lyapunov Exponent, when calculated using the Rosenstein method, implies that the trajectories of the system converge over time. This convergence suggests that the system is stable, and its future behavior is predictable over the long term [50]. In practical terms, minor differences in initial states will diminish over time, leading to similar outcomes or states. This stability is characteristic of non-chaotic systems, where, despite slight variations at the start, the system evolves predictably without sensitive dependence on those initial conditions.

In Table 5, regarding the ANN prediction errors, the ApEn has the highest error for the COM velocity (29.306%) and the lowest for the hip velocity (14.893%), indicating varying degrees of predictability across gait parameters. The SpEn’s prediction error is greatest for the GRF_Ver_ (33.525%) and lowest for the hip velocity (9.863%), suggesting that vertical force predictions are less reliable. The CD model’s error is highest for the ankle moment (38.107%) and lowest for the GRF_Ver_ (12.112%), aligning with the complexity noted in CD values. DFA models have the most substantial prediction error for the COM velocity (51.333%) and the smallest for the knee angle (10.430%), indicating a challenge in accurately predicting the metabolic cost based on COM movement. Errors from the LyE_w_ model are most significant for the ankle velocity (24.799%) and least for the hip velocity (17.627%), while the LyE_R_ model shows the highest error for the hip angle (15.897%) and an impressively low error for the COM velocity (5.834%), which is interesting considering its negative value in nonlinearity measures. These prediction errors are crucial for understanding the reliability of each nonlinearity measure in estimating the metabolic cost and can guide the development of more sophisticated models for gait analysis.

In analyzing the influence of various biomechanical variables on the prediction of the metabolic cost using ANN models, as outlined in Table 5, certain variables stand out due to their higher prediction errors across different nonlinearity measures. For instance, the ankle velocity and COM velocity demonstrate significant prediction errors (ApEn, LyE_w_, and SpEn for the ankle velocity and the ApEn and DFA for the COM velocity), underscoring their critical influence in the models. ApEn errors suggest that the model struggles with the complexity and adaptability of the ankle and COM velocity, as the ankle velocity is essential for propulsion and balance, and the COM velocity requires frequent adjustments for stability. LyEw errors for the ankle velocity indicate potential challenges in capturing sensitivity to small perturbations, contributing to nonlinear variability. High SpEn errors point to the irregularity in ankle force generation needed for propulsion. In contrast, DFA errors in the COM velocity reflect the complexity of long-range correlations required for balance control. These errors highlight the intricate biomechanical interactions these variables represent, as well as the modeling challenges involved.

Additionally, the GRF_Ver_ exhibits a high prediction error in DFA, highlighting its importance in capturing the complex gait dynamics. The ankle moment also shows a substantial prediction error in the CD, indicating its significant role in the nonlinear dynamics associated with the metabolic cost. The high prediction error for the GRF_Ver_ in DFA suggests that the vertical ground reaction force plays a key role in capturing long-term dependencies in gait patterns, as it directly impacts each step’s stability and energy requirements, especially in maintaining a balance against gravitational forces. Similarly, the substantial prediction error for the ankle moment in the CD highlights the complexity of the ankle torque in contributing to the multidimensional aspects of gait dynamics. Ankle moments are critical for propulsion and stabilization, and their variability reflects the intricate control required for efficient energy use, which is closely tied to the metabolic cost.

These findings suggest that the ankle velocity, COM velocity, GRF_Ver_, and ankle moment are key factors in the ANN models’ predictive capabilities, especially in the context of the nonlinear characteristics of gait and their impact on metabolic efficiency. Table 5 shows that the highest mean error of the nets comes from the GRF, and the lowest comes from the knee. It also shows that, across the gait parameters, the COM velocity, GRF_ver_, knee velocity, ankle moment, and hip angle influence the ANN-based metabolic cost predictions the most. Consistent with existing research [51,52], it is indicated that factors such as the GRF, COM dynamics, moments at various joints, and how mechanical work is shared among the hip, knee, and ankle joints play crucial roles in influencing the metabolic cost of movement.

In summary, the tables and figure analysis suggest that the LyE_R_ and DFA show the highest explanatory power for the metabolic cost among the nonlinear gait measures, which is aligned with the literature [53–58]. Specifically, the R^2^_MLR_ values from the multiple linear regression analyses indicate that DFA and the LyE_R_ can explain 63% and 72% of the variance in the metabolic cost, respectively. Among the ANN models, models created based on the LyE_R_, DFA, and ApEn showed better performance, as indicated by their low MSE and high R^2^_ANN_ values. This suggests that these measures capture variations in gait that are more closely related to the metabolic cost than others. The coefficients (estimates) reflect the change in the metabolic cost associated with a one-unit change in the variable and help elucidate the most influential variables within the MLR and ANN models. Variables with significant *p*-values are considered strong predictors of the metabolic cost. Although the specifics of these variables are not quoted directly here, the high R^2^_ANN_ values for the DFA and LyE_R_ models suggest that the variables included in these models are substantial connectors to the predicted metabolic cost. Based on the R^2^_ANN_ values, DFA and the LyE_R_ appear to predict the greatest proportion of the metabolic costs. Within the ANN and MLR models, the specific variables that most effectively change the metabolic cost would be those with significant coefficients in the models using DFA and LyE_R_ measures. It is recommended that these variables be focused on optimizing gait parameters for metabolic cost reduction in assistive devices such as hip exoskeletons.

The integration of AI in healthcare introduces critical ethical considerations, such as data privacy, algorithmic biases, and the transparency of AI decision-making. Public perspectives on AI are mixed, reflecting concerns about trustworthiness and data misuse alongside optimism for enhanced diagnostics and personalized care. Recent studies emphasize the need for robust regulatory frameworks and stakeholder engagement to ensure AI is deployed ethically and responsibly in clinical settings, particularly for applications like gait analysis and assistive technologies [30–33].

While our findings demonstrate the potential of ANN models in predicting metabolic costs associated with nonlinear gait dynamics, these results should be considered preliminary. A primary limitation is the small sample size, which may restrict the generalizability of the findings to broader populations. The k-fold cross-validation and regularization approaches were applied to reduce this risk. It also may reduce statistical power, increasing the potential for Type II errors and limiting the generalizability of results. Future studies with larger and more varied cohorts would be beneficial for validating and expanding upon these findings (e.g., integrating multi-planar analyses for a more comprehensive view of gait dynamics). Although the dataset consists of 10 participants, each trial includes multiple strides, resulting in a larger dataset that increases statistical power. Although this approach has been used in previous studies [10,14], this structure may amplify the significance of small effects, contributing to the low *p*-values reported. While this enhances the ability to detect patterns, it is important to interpret these results cautiously, given the relatively small number of participants [10,14]. The study’s focus on specific nonlinear measures and ANN models could also introduce biases, potentially overlooking other significant variables influencing the metabolic cost during gait. Future studies could explore other machine learning models to assess their effectiveness in predicting the metabolic cost in gait analysis and potentially enhance model performance. In addition, for future studies, we suggest exploring alternative optimization methods for hyperparameter tuning in ANN models, such as Bayesian optimization [10]. Moreover, the participant group’s homogeneity, consisting solely of healthy young adults, further limits the applicability of our findings to diverse populations, such as older adults or individuals with gait impairments. Future studies should also explore model performance across different gait conditions, such as varying walking speeds and running and treadmill walking vs. overground walking, to enhance the model’s generalizability and adaptability to broader real-world scenarios. Safety is critical when applying ANNs in assistive technologies, especially in unpredictable environments. ANN models could be implemented alongside monitoring systems that detect deviations from expected gait patterns and/or anatomically safe movements, thus allowing for real-time adjustments to mitigate risks. The experimental conditions, mainly using the hip exoskeleton and defined walking tasks, might not encapsulate the complexity and variability inherent in everyday walking scenarios. This limitation could affect the study’s external validity, as the findings might not be directly extrapolatable to real-world gait dynamics outside of controlled laboratory environments. Future work should validate the model with newly acquired or publicly available datasets.

## 5. Conclusions

The study explored the intricate relationship between gait nonlinearities and the metabolic cost, hypothesizing that specific nonlinear gait parameters significantly influence the metabolic cost during walking. The research utilized advanced analytical models such as ANNs and MLR to decipher the complex dynamics of human gait, mainly focusing on variables such as the ankle velocity, COM velocity, ground reaction forces, and joint moments. The hypothesis was grounded in the premise that a deeper understanding of these nonlinear dynamics could enhance the predictive accuracy of the metabolic cost, thereby offering novel insights into gait optimization, especially for individuals using assistive devices like hip exoskeletons. In conclusion, the study’s findings robustly support the initial hypothesis, revealing that DFA and the LyE_R_ exhibit the highest explanatory power for the metabolic cost among the array of nonlinear gait measures examined. The R^2^_MLR_ values obtained from the multiple linear regression analyses—72% for the LyE_R_ and 63% for DFA—underscore the significant variance in the metabolic cost these measures can explain. ANN models tailored to the LyE_R_, DFA, and ApEn outperformed others, showcasing low MSE and high R^2^_ANN_ values. This superior performance suggests these models’ efficacy in capturing the variability in gait that correlates closely with the metabolic cost. The ANN models developed in this study offer a unique advantage over traditional approaches by enabling potential real-time implementation alongside monitoring systems. By detecting deviations from expected gait patterns, these models could facilitate immediate adjustments to gait parameters, thus mitigating the risk of injury or strain. This adaptability provides a foundation for innovative applications in personalized rehabilitation and assistive device optimization, making our approach distinct in its ability to support safe, individualized gait interventions. The study recommends a focused examination of the variables with significant coefficients within these models, as they are potent predictors of the metabolic cost and pivotal in optimizing gait parameters for metabolic efficiency in assistive devices like hip exoskeletons. Our results offer valuable insights for exoskeleton robot design, especially optimizing control strategies. By accurately predicting the metabolic cost and key gait parameters, the ANN models can guide real-time adjustments in the torque, alignment, and support, reducing user fatigue, enhancing stability, and enabling more responsive, individualized gait assistance for safer and more effective rehabilitation.

## Supplementary Material

S

**Supplementary Materials:** The following supporting information can be downloaded at https://www.mdpi.com/article/10.3390/app142311026/s1: Figure S1: Comprehensive regression analysis of the net_LyER_ model; Figure S2: Comprehensive regression analysis of the net_DFA_ model; Figure S3: Comprehensive regression analysis of the net_ApEn_ model; Figure S4: Comprehensive regression analysis of the net_CD_ model; Figure S5: Comprehensive regression analysis of the _SpEn_ model; Figure S6: Comprehensive regression analysis of the _LyEW_ model; Figure S7: Error histograms for the nonlinear models. Figure S8: Comprehensive Regression Analysis of the Multiple Linear Regression Models (MLR). Figure S9: Error Histograms for the Multiple Linear Regression Models (MLR). Table S1: 95% Confidence Intervals (CI) for Multiple Linear Regression (MLR) coefficients and Artificial Neural Network (ANN) models across six different gait nonlinearity measures.

## Figures and Tables

**Table 1.** Listing all measured signals and their corresponding normalization processes.

Signal Type	Measurement	Normalization Process

Joint Angle	Hip, Knee, Ankle	N/A
Joint Velocity	Hip, Knee, Ankle	N/A
Joint Moment	Hip, Knee, Ankle	Normalized to body mass (N·m/kg)
Center of Mass	Position, Velocity	N/A
Ground Reaction Force	Anterior-Posterior and Vertical Components	Normalized to body mass (N/kg)
Metabolic Cost	Indirect Calorimetry	Normalized to body mass (W/kg)

This table presents signals and their corresponding normalization processes. Joint moments and the GRF were normalized to each participant’s body mass (N·m/kg for moments and N/kg for the GRF), while the metabolic cost was expressed in watts per kilogram (W/kg) to allow for meaningful cross-trial and cross-condition comparisons, minimizing individual variability and facilitating the analysis. “N/A” indicates that the value is not applicable or could not be calculated for the corresponding metric.

**Table 2.** Performance metrics of Artificial Neural Network (ANN) models for gait nonlinearity measures.

	LyE_R_	DFA	ApEn	CD	SpEn	LyE_w_

**Validation ratio (%)**	15	15	15	15	15	15
**Test ratio (%)**	15	15	15	15	15	15
**Training ratio (%)**	70	70	70	70	70	70
**Hidden layer size (neurons)**	8	19	14	3	18	2

**MSE performance (W/kg)^2^:**						
- Validation	50 × 10^−6^	40 × 10^−6^	40 × 10^−6^	60 × 10^−6^	90 × 10^−6^	60 × 10^−5^
- Test	30 × 10^−6^	20 × 10^−6^	70 × 10^−6^	17 × 10^−5^	13 × 10^−5^	37 × 10^−5^
- Training	10 × 10^−6^	20 × 10^−6^	90 × 10^−6^	10 × 10^−6^	10 × 10^−5^	10 × 10^−6^
- Total	20 × 10^−6^	20 × 10^−6^	20 × 10^−6^	10 × 10^−5^	50 × 10^−6^	22 × 10^−5^
- 5-Fold	41 × 10^−6^ ± 8 × 10^−6^	45 × 10^−6^ ± 15 × 10^−6^	112 × 10^−6^ ± 23 × 10^−6^	262 × 10^−6^ ± 89 × 10^−6^	116 × 10^−6^ ± 21 × 10^−6^	428 × 10^−6^ ± 48 × 10^−6^

**R^2^_ANN_:**						
- Validation	0.94	0.94	0.92	0.85	0.91	0.63
- Test	0.92	0.97	0.90	0.85	0.86	0.64
- Training	0.98	0.96	0.86	0.98	0.79	0.98
- Total	0.97	0.97	0.96	0.85	0.92	0.62
- 5-Fold	0.97 ± 0.01	0.97 ± 0.02	0.94 ± 0.02	0.90 ± 0.03	0.93 ± 0.03	0.74 ± 0.06

***p*-value:**						
- Validation	<0.0001	<0.0001	<0.0001	<0.0001	<0.0001	<0.0001
- Test	<0.0001	<0.0001	<0.0001	<0.0001	<0.0001	<0.0001
- Training	<0.0001	<0.0001	<0.0001	<0.0001	<0.0001	<0.0001
- Total	<0.0001	<0.0001	<0.0001	<0.0001	<0.0001	<0.0001
-5-Fold	<0.0001	<0.0001	<0.0001	<0.0001	<0.0001	<0.0001

This table summarizes the training, validation, and test ratios, the number of neurons in the hidden layer, the mean squared error (MSE) performance across training, validation, and testing phases, R^2^_ANN_ values indicating the models’ fit, and *p*-values for statistical significance, as well as a k-fold cross-validation test with k = 5 (5-fold), across six different ANN models corresponding to the following gait nonlinearity measures: the Lyapunov Exponent based on Rosenstein’s algorithm (LyE_R_), Detrended Fluctuation Analysis (DFA), the Approximate Entropy (ApEn), the correlation dimension (CD), the Sample Entropy (SpEn), and the Lyapunov Exponent based on Wolf’s algorithm (LyEW). The “Total” R- and *p*-values are calculated using all data combined across the training, validation, and test phases rather than representing an average or summation. This approach provides an overall measure of the model’s performance across all data, offering a comprehensive assessment of the ANN’s predictive accuracy.

**Table 3.** Summary of multiple linear regression (MLR) analysis for gait nonlinearity measures.

		LyE_R_			DFA			ApEn	

Coefficient of Determination (R^2^_MLR_)		0.72			0.63			0.46	

Variable	Estimate	SE (W/Kg)	*p*-value	Estimate	SE (W/Kg)	*p*-value	Estimate	SE (W/Kg)	*p*-value

β0 (Intercept)	−0.0557	0.0284	0.0530	0.3161	0.1885	0.0966	**0.1504**	**0.0337**	**<0.0001**
β1 (Hip angle)	−0.3943	0.6463	0.5431	−0.0554	0.0760	0.4676	−0.0182	0.1552	0.9071
β2 (Hip velocity)	**1.2900**	**0.3324**	**0.0002**	−0.1441	0.0777	0.0665	−0.1028	0.0956	0.2846
β3 (Hip moment)	**−1.7930**	**0.4806**	**0.0003**	**−0.3464**	**0.0995**	**0.0007**	**0.1744**	**0.0570**	**0.0028**
β4 (Knee angle)	0.7355	0.3812	0.0564	−0.0494	0.0904	0.5856	0.1635	0.1219	0.1827
β5 (Knee velocity)	**−1.3390**	**0.6619**	**0.0456**	**−0.4148**	**0.1242**	**0.0012**	−0.0933	0.1056	0.3790
β6 (Knee moment)	**0.3640**	**0.1349**	**0.0081**	−0.0607	0.0361	0.0952	−0.0692	0.0370	0.0643
β7 (Ankle angle)	**−1.3730**	**0.3857**	**0.0006**	**−0.1688**	**0.0527**	**0.0018**	0.0887	0.0488	0.0719
β8 (Ankle velocity)	**2.7300**	**0.4224**	**<0.0001**	**0.3245**	**0.0599**	**<0.0001**	**−0.2695**	**0.0559**	**<0.0001**
β9 (Ankle moment)	0.7046	0.4625	0.1306	**0.4847**	**0.1133**	**<0.0001**	0.0631	0.0661	0.3414
β10 (GRF Ver)	**0.7823**	**0.1380**	**<0.0001**	**−0.4842**	**0.1862**	**0.0106**	**0.3785**	**0.1032**	**0.0004**
β11 (GRF AP)	**−1.8880**	**0.4286**	**<0.0001**	−0.1595	0.1949	0.4149	**−0.4155**	**0.1119**	**0.0003**
β12 (COM position)	**3.1730**	**1.3410**	**0.0198**	−0.0796	0.0424	0.0634	0.0341	0.1212	0.7789
β13 (COM velocity)	−2.2800	2.1580	0.2931	**1.1820**	**0.2669**	**<0.0001**	−0.1241	0.1215	0.3096

This table presents the results of multiple linear regression analyses for the Lyapunov Exponent based on Rosenstein’s algorithm (LyE_R_), Detrended Fluctuation Analysis (DFA), and the Approximate Entropy (ApEn) as predictors of the metabolic cost. The table includes the coefficient of determination (R^2^_MLR_) values, which indicate the proportion of variance in the metabolic cost that is predictable from the nonlinearity measures. Regression coefficients (β values = estimates), Standard Errors (SE), and *p*−values are listed for each predictor variable, including joint angles, velocities, moments, ground reaction forces (GRFs), and the center of mass (COM) position and velocity, providing insight into their statistical significance and impact on the predicted metabolic cost. Values with statistically significant *p*-values are highlighted in bold font.

**Table 4.** Summary of multiple linear regression (MLR) analysis for gait nonlinearity measures.

		CD			SpEn			LyE_w_	

Coefficient of Determination (R^2^_MLR_)		0.42			0.41			0.25	

Variable	Estimate	SE (W/Kg)	*p*-value	Estimate	SE (W/Kg)	*p*-value	Estimate	SE (W/Kg)	*p*-value

β0 (Intercept)	0.2221	0.1192	0.0652	0.0391	0.0449	0.3853	**0.0491**	**0.0077**	**<0.0001**
β1 (Hip angle)	0.0358	0.0375	0.3421	−0.0440	0.1040	0.6733	0.0039	0.0043	0.3687
β2 (Hip velocity)	−0.0344	0.0395	0.3853	**−0.2262**	**0.0662**	**0.0009**	−0.0030	0.0085	0.7245
β3 (Hip moment)	0.0553	0.0440	0.2120	**0.1801**	**0.0538**	**0.0011**	−0.0006	0.0034	0.8569
β4 (Knee angle)	0.0105	0.0425	0.8048	0.0864	0.0786	0.2744	−0.0198	0.0108	0.0688
β5 (Knee velocity)	−0.0765	0.0399	0.0577	0.0935	0.0787	0.2375	**0.0339**	**0.0106**	**0.0019**
β6 (Knee moment)	−0.0246	0.0282	0.3846	**−0.0992**	**0.0382**	**0.0107**	−0.0009	0.0030	0.7668
β7 (Ankle angle)	−0.0448	0.0297	0.1340	−0.0809	0.0460	0.0816	−0.0047	0.0074	0.5249
β8 (Ankle velocity)	−0.0557	0.0374	0.1388	−0.0547	0.0437	0.2133	**0.0199**	**0.0082**	**0.0168**
β9 (Ankle moment)	**0.0827**	**0.0337**	**0.0156**	0.1742	0.1519	0.2539	−0.0010	0.0027	0.7053
β10 (GRF Ver)	**−0.9418**	**0.2991**	**0.0021**	**0.6748**	**0.3341**	**0.0460**	0.0002	0.0006	0.7892
β11 (GRF AP)	**0.1864**	**0.0871**	**0.0346**	**−0.7148**	**0.1911**	**0.0003**	−0.0036	0.0055	0.5216
β12 (COM position)	0.0127	0.0290	0.6623	−0.1050	0.0788	0.1858	**−0.0281**	**0.0086**	**0.0015**
β13 (COM velocity)	−0.0122	0.0361	0.7354	0.2258	0.1240	0.0714	0.0081	0.0075	0.2793

This table presents the results of multiple linear regression analyses for the correlation dimension (CD), Sample Entropy (SpEn), and Lyapunov Exponent based on Wolf’s algorithm (LyE_w_) as predictors of the metabolic cost. The table includes the coefficient of determination (R^2^_MLR_) values, which indicate the proportion of variance in the metabolic cost that is predictable from the nonlinearity measures. Regression coefficients (β values = estimates), Standard Errors (SE), and *p*-values are listed for each predictor variable, including joint angles, velocities, moments, ground reaction forces (GRFs), and the center of mass (COM) position and velocity, providing insight into their statistical significance and impact on the predicted metabolic cost. Values with statistically significant *p*-values are highlighted in bold font.

**Table 5.** Summary of nonlinear gait parameters and ANN prediction errors.

Summary of Figure 2	Hip Angle	Hip Velocity	Hip Moment	Knee Angle	Knee Velocity	Knee Moment	Ankle Angle	Ankle Velocity	Ankle Moment	GRF Ver	GRF AP	COM Position	COM Velocity

**LyE_R_**	0.049	0.038	0.038	0.034	0.022	0.025	0.037	0.020	0.060	0.271	0.059	−0.001	−0.003
**DFA**	0.780	0.671	0.684	0.653	0.515	0.621	0.665	0.446	0.677	0.691	0.635	0.553	0.444
**ApEn**	0.240	0.269	0.346	0.250	0.348	0.369	0.282	0.390	0.233	0.217	0.222	0.319	0.289
**CD**	1.733	1.838	1.843	1.754	1.781	1.774	1.794	1.861	1.201	0.075	0.292	1.883	1.888
**SpEn**	0.154	0.216	0.242	0.191	0.287	0.268	0.224	0.301	0.103	0.125	0.113	0.321	0.284
**LyE_W_**	0.745	1.028	1.669	0.792	0.883	1.765	1.004	1.193	2.068	1.570	1.233	0.712	0.973

**Error net LyE_R_ (%)**	15.897	10.573	6.931	6.288	8.031	6.538	8.212	7.432	12.102	14.650	10.329	5.975	5.834
**Error net DFA (%)**	25.801	28.735	26.493	10.430	29.352	12.471	15.335	15.122	27.540	49.067	34.960	10.521	51.333
**Error net ApEn (%)**	19.973	14.893	14.883	13.192	41.522	13.981	11.535	24.361	15.188	17.604	18.660	26.751	29.306
**Error net CD (%)**	15.519	11.798	16.745	13.275	13.911	19.324	14.212	12.957	38.107	12.112	21.554	13.397	16.126
**Error net SpEn (%)**	11.004	9.863	11.091	10.866	16.947	12.213	10.098	19.340	13.541	33.525	17.096	13.524	15.305
**Error net LyE_W_ (%)**	17.692	17.627	17.737	16.972	17.765	16.866	17.881	24.799	17.517	17.664	18.495	17.989	18.625

**Mean error Nets (%)**	17.648	15.582	15.647	11.837	21.255	13.566	12.879	17.335	20.666	24.104	20.182	14.693	22.755

		16.292			15.553			16.960		22.143		18.724

This table compares the mean of nonlinear measures across various gait parameters, including hip, knee, and ankle angles, velocities, moments, ground reaction forces (GRFs), and center of mass (COM) dynamics. The nonlinearity measures are Lyapunov Exponents based on Rosenstein’s algorithm (LyE_R_), Detrended Fluctuation Analysis (DFA), the Approximate Entropy (ApEn), the correlation dimension (CD), the Sample Entropy (SpEn), and Lyapunov Exponents based on Wolf’s algorithm (LyE_w_). We explore each variable of interest’s impact on the predicted outcome by varying it across its entire range while keeping all other variables constant. This process involves assessing the model’s predictions at several points within this range to understand how changes in the variable affect the outcome. This method allows us to pinpoint the variable’s influence on the model’s predictions. Additionally, the table lists the percentage of prediction error for each nonlinearity measure as determined by ANN models, offering insights into the predictive reliability of each measure for the metabolic cost during gait.

## Data Availability

The raw data supporting the conclusions of this article will be made available by the authors upon request.
