# Supplementary material for: How Gait Nonlinearities in Individuals Without Known Pathology Describe Metabolic Cost During Walking Using Artificial Neural Network and Multiple Linear Regression
Source: Appl Sci (Basel). Author manuscript; Available in PMC 2026 Aug 28. (PMC13520628; doi:10.3390/app142311026)
Supplement: Supplementary file 1 [file NIHMS2202043-supplement-S.zip › Supplementary Materials_94.docx]

**Supplementary data**

s8

**Figure S1 -** **Comprehensive Regression Analysis of the net_LyER_ Model.** This figure delineates the regression plots for the net_LyER_ model across A) 'validation', B) 'test', C) 'training', and D) 'all' datasets, illustrating the model's predictive accuracy and generalization capability. The plots compare the predicted metabolic costs against actual values, with each subset providing insights into the model's performance in different phases of data handling, from learning to generalization on unseen data.

**Figure S2 -** **Comprehensive Regression Analysis of the net_DFA_ Model.** This figure delineates the regression plots for the net_DFA_ model across A) 'validation', B) 'test', C) 'training', and D) 'all' datasets, illustrating the model's predictive accuracy and generalization capability. The plots compare the predicted metabolic costs against actual values, with each subset providing insights into the model's performance in different phases of data handling, from learning to generalization on unseen data.

**Figure S3 -** **Comprehensive Regression Analysis of the net_ApEn_ Model.** This figure delineates the regression plots for the net_ApEn_ model across A) 'validation', B) 'test', C) 'training', and D) 'all' datasets, illustrating the model's predictive accuracy and generalization capability. The plots compare the predicted metabolic costs against actual values, with each subset providing insights into the model's performance in different phases of data handling, from learning to generalization on unseen data.

**Figure S4 -** **Comprehensive Regression Analysis of the net_CD_ Model.** This figure delineates the regression plots for the net_CD_ model across A) 'validation', B) 'test', C) 'training', and D) 'all' datasets, illustrating the model's predictive accuracy and generalization capability. The plots compare the predicted metabolic costs against actual values, with each subset providing insights into the model's performance in different phases of data handling, from learning to generalization on unseen data.

**Figure S5 -** **Comprehensive Regression Analysis of the _SpEn_ Model.** This figure delineates the regression plots for the net_SpEn_ model across A) 'validation', B) 'test', C) 'training', and D) 'all' datasets, illustrating the model's predictive accuracy and generalization capability. The plots compare the predicted metabolic costs against actual values, with each subset providing insights into the model's performance in different phases of data handling, from learning to generalization on unseen data.

**Figure S6 -** **Comprehensive Regression Analysis of the _LyEW_ Model.** This figure delineates the regression plots for the net_LyEW_ model across A) 'validation', B) 'test', C) 'training', and D) 'all' datasets, illustrating the model's predictive accuracy and generalization capability. The plots compare the predicted metabolic costs against actual values, with each subset providing insights into the model's performance in different phases of data handling, from learning to generalization on unseen data.

| **(A)** | **(B)** |
| --- | --- |
|  |  |
| **(C)** | **(D)** |
|  |  |
| **(E)** | **(F)** |
|  |  |
| **Figure S7 -** **Error Histograms for the nonlinear Models.** Each panel presents the error distribution for **A)** net_LyER_, **B)** net_DFA_, **C)** net_ApEn_, **D)** net_CD_, **E)** net_SpEn_, and **F)** net_LyEw_. They illustrate the variance between predicted and actual metabolic costs. The histograms elucidate the frequency of error magnitudes, aiding in identifying consistent prediction deviations and outlier errors for model refinement. A concentration of errors closer to zero for the ANN models indicates a tighter clustering of predicted values around the actual metabolic costs and, consequently, a higher accuracy. In contrast, a broader spread of errors for the ANN models implies a greater variance in the accuracy of predictions. | |

| **(A)** | **(B)** |
| --- | --- |
|  |  |
| **(C)** | **(D)** |
|  |  |
| **(E)** | **(F)** |
|  |  |
| **Figure S8 - Comprehensive Regression Analysis of the Multiple Linear Regression Models (MLR).** Each panel delineates the regression plots for the MLR models across **A)** MLR_LyER_, **B)** MLR_DFA_, **C)** MLR_ApEn_, **D)** MLR_CD_, **E)** MLR_SpEn_, and **F)** MLR_LyEw_, illustrating the model's predictive accuracy and generalization capability. The plots compare the predicted metabolic costs against actual values, providing insights into the model's performance. | |

| **(A)** | **(B)** |
| --- | --- |
|  |  |
| **(C)** | **(D)** |
|  |  |
| **(E)** | **(F)** |
|  |  |
| **Figure S9 - Error Histograms for the Multiple Linear Regression Models (MLR).** Each panel presents the error distribution for **A)** MLR_LyER_, **B)** MLR_DFA_, **C)** MLR_ApEn_, **D)** MLR_CD_, **E)** MLR_SpEn_, and **F)** MLR_LyEw_. They illustrate the variance between predicted and actual metabolic costs. The histograms elucidate the frequency of error magnitudes, aiding in identifying consistent prediction deviations and outlier errors for model refinement. A concentration of errors closer to zero for the MLR models indicates a tighter clustering of predicted values around the actual metabolic costs and, consequently, a higher accuracy. In contrast, a broader spread of errors for the MLR models implies a greater variance in the accuracy of predictions. | |

| **Table S1.** 95% Confidence Intervals (CI) for Multiple Linear Regression (MLR) coefficients and Artificial Neural Network (ANN) models across six different gait nonlinearity measures: Lyapunov Exponent based on Rosenstein's algorithm (LyE_R_), Detrended Fluctuation Analysis (DFA), Approximate Entropy (ApEn), Correlation Dimension (CD), Sample Entropy (SpEn), and Lyapunov Exponent based on Wolf's algorithm (LyE_W_). Each variable represents a specific gait parameter, including joint angles, velocities, moments, ground reaction forces (GRF), and center of mass (COM) metrics. The lower and upper bounds of the CI indicate the range within which the true effect of each variable is likely to lie for each gait nonlinearity measure. | | | | | | | | | | | | |
| --- | --- | --- | --- | --- | --- | --- | --- | --- | --- | --- | --- | --- |
| **95% Confidence**  **Intervals (CI)** | **LyE_R_** | | **DFA** | | **ApEn** | | **CD** | | **SpEn** | | **LyE_w_** | |
|  | **Lower** | **Upper** | **Lower** | **Upper** | **Lower** | **Upper** | **Lower** | **Upper** | **Lower** | **Upper** | **Lower** | **Upper** |
| **MLR Variable** |  |  |  |  |  |  |  |  |  |  |  |  |
| β0 (Intercept) | -0.1120 | 0.0007 | -0.0577 | 0.6899 | 0.0836 | 0.2172 | -0.0143 | 0.4584 | -0.0499 | 0.1281 | 0.0338 | 0.0643 |
| β1 (Hip angle) | -1.6760 | 0.8871 | -0.2061 | 0.0953 | -0.3259 | 0.2896 | -0.0385 | 0.1100 | -0.2502 | 0.1622 | -0.0046 | 0.0123 |
| β2 (Hip velocity) | 0.6306 | 1.9490 | -0.2981 | 0.0100 | -0.2923 | 0.0867 | -0.1127 | 0.0439 | -0.3575 | -0.0949 | -0.0199 | 0.0139 |
| β3 (Hip moment) | -2.7450 | -0.8396 | -0.5437 | -0.1492 | 0.0614 | 0.2875 | -0.0320 | 0.1426 | 0.0734 | 0.2868 | -0.0073 | 0.0061 |
| β4 (Knee angle) | -0.0204 | 1.4910 | -0.2286 | 0.1297 | -0.0782 | 0.4052 | -0.0737 | 0.0948 | -0.0695 | 0.2422 | -0.0411 | 0.0016 |
| β5 (Knee velocity) | -2.6510 | -0.0268 | -0.6610 | -0.1686 | -0.3026 | 0.1160 | -0.1556 | 0.0025 | -0.0625 | 0.2495 | 0.0128 | 0.0550 |
| β6 (Knee moment) | 0.0965 | 0.6314 | -0.1322 | 0.0108 | -0.1425 | 0.0042 | -0.0806 | 0.0313 | -0.1750 | -0.0235 | -0.0068 | 0.0050 |
| β7 (Ankle angle) | -2.1380 | -0.6081 | -0.2732 | -0.0644 | -0.0080 | 0.1855 | -0.1036 | 0.0140 | -0.1721 | 0.0103 | -0.0193 | 0.0099 |
| β8 (Ankle velocity) | 1.8920 | 3.5670 | 0.2057 | 0.4434 | -0.3803 | -0.1586 | -0.1298 | 0.0184 | -0.1414 | 0.0319 | 0.0037 | 0.0361 |
| β9 (Ankle moment) | -0.2124 | 1.6220 | 0.2601 | 0.7094 | -0.0679 | 0.1941 | 0.0160 | 0.1495 | -0.1269 | 0.4754 | -0.0063 | 0.0043 |
| β10 (GRF Ver) | 0.5088 | 1.0560 | -0.8534 | -0.1150 | 0.1739 | 0.5832 | -1.5350 | -0.3487 | 0.0124 | 1.3370 | -0.0011 | 0.0015 |
| β11 (GRF AP) | -2.7380 | -1.0380 | -0.5458 | 0.2268 | -0.6374 | -0.1936 | 0.0137 | 0.3591 | -1.0940 | -0.3359 | -0.0146 | 0.0074 |
| β12 (COM position) | 0.5149 | 5.8320 | -0.1637 | 0.0045 | -0.2061 | 0.2743 | -0.0448 | 0.0702 | -0.2613 | 0.0513 | -0.0452 | -0.0110 |
| β13 (COM velocity) | -6.5580 | 1.9980 | 0.6525 | 1.7110 | -0.3649 | 0.1168 | -0.0837 | 0.0593 | -0.0201 | 0.4717 | -0.0067 | 0.0229 |
| **ANN Model** | 0.0000 | 0.0001 | 0.0000 | 0.0001 | 0.0001 | 0.0002 | 0.0001 | 0.0004 | 0.0001 | 0.0002 | 0.0003 | 0.0005 |
